# Dissection of the region of *Pseudomonas aeruginosa* ParA that is important for dimerization and interactions with its partner ParB

**DOI:** 10.1099/mic.0.081216-0

**Published:** 2014-11

**Authors:** Aneta A. Bartosik, Krzysztof Glabski, Paulina Jecz, Krzysztof Lasocki, Malgorzata Mikosa, Danuta Plochocka, Christopher M. Thomas, Grazyna Jagura-Burdzy

**Affiliations:** 1 Institute of Biochemistry and Biophysics, Polish Academy of Sciences, Pawinskiego 5A, 02-106 Warsaw, Poland; 2 School of Biosciences, University of Birmingham, Edgbaston, Birmingham B15 2TT, UK

## Abstract

*Pseudomonas aeruginosa* ParA belongs to a large subfamily of Walker-type ATPases acting as partitioning proteins in bacteria. ParA has the ability to both self-associate and interact with its partner ParB. Analysis of the deletion mutants defined the part of the protein involved in dimerization and interactions with ParB. Here, a set of ParA alanine substitution mutants in the region between E67 and L85 was created and analysed *in vivo* and *in vitro*. All mutants impaired in dimerization (substitutions at positions M74, H79, Y82 and L84) were also defective in interactions with ParB, suggesting that ParA–ParB interactions depend on the ability of ParA to dimerize. Mutants with alanine substitutions at positions E67, C68, L70, E72, F76, Q83 and L85 were not impaired in dimerization, but were defective in interactions with ParB. The dimerization interface partly overlapped the pseudo-hairpin, involved in interactions with ParB. ParA mutant derivatives tested *in vitro* showed no defects in ATPase activity. Two *parA* alleles (*parA84*, whose product can neither self-interact nor interact with ParB, and *parA67*, whose product is impaired in interactions with ParB, but not in dimerization) were introduced into the *P. aeruginosa* chromosome by homologous gene exchange. Both mutants showed defective separation of ParB foci, but to different extents. Only PAO1161 *parA84* was visibly impaired in terms of chromosome segregation, growth rate and motility, similar to a *parA*-null mutant.

## Introduction

The faithful segregation of low-copy-number plasmids depends on the plasmid-specific partition complex of two Par proteins and a centromere-like sequence *parS*. Despite the variety of plasmid partitioning systems, there are common features: component A, an NTPase (Walker-type ATPase, actin-type ATPase, tubulin-like GTPase), forms the dynamic scaffold for plasmid movement to the progeny cells (Ah-Seng *et al.*, 2013; Vecchiarelli *et al.*, 2010), whereas component B, a DNA-binding protein, recognizes and binds *parS*, forming the segregating unit – the segrosome (Barillà & Hayes, 2003; Gerdes *et al.*, 2010; Pratto *et al.*, 2008). Interactions between these two proteins stimulate NTP hydrolysis and lead the movement of segrosomes over the nucleoid (Gerdes *et al.*, 2010).

**Fig. 1.**
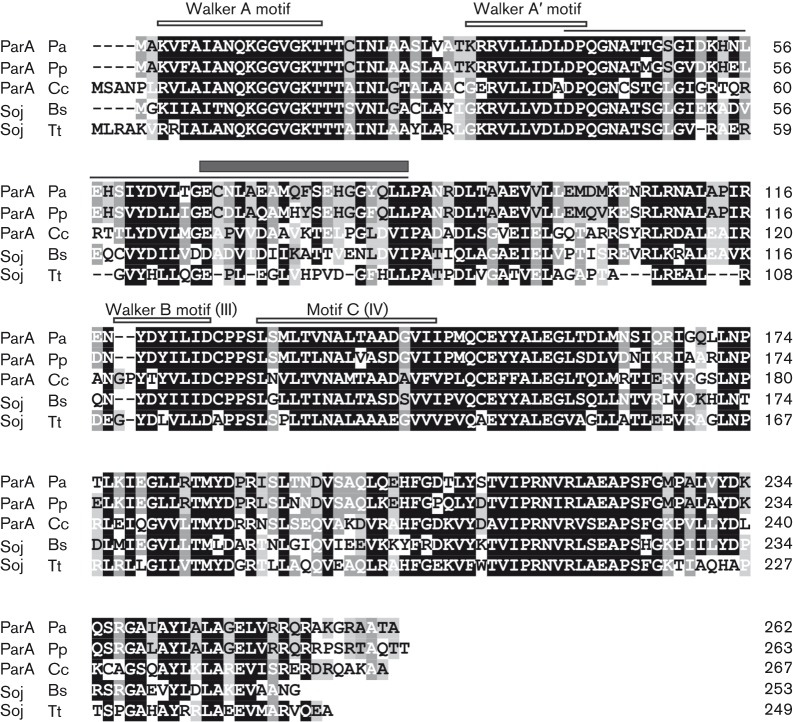
Comparison of the best-studied chromosomal ParA family members. Amino acids similar in at least four proteins are marked by a black background, those similar in three are marked by a dark grey background and homologous residues in two proteins are marked by a light grey background. Conserved ATP-binding Walker A, A′ and B (III) motifs (Koonin, 1993) and motif C (IV) characteristic of ParA-like proteins are indicated. The *P. aeruginosa* ParA region defined by deletion mapping is shown by a thin line above the sequence and the region analysed by alanine scanning is shown by a dark grey box. Pa, *Pseudomonas aeruginosa*; Pp, *Pseudomonas putida*; Cc, Caulobacter *crescentus*; Bs, *Bacillus subtilis*; Tt, *Thermus thermophilus*.

The homologues of class I plasmidic Par proteins, encoded on bacterial chromosomes in close proximity to the origin of replication, form their own subgroups (Gerdes *et al.*, 2000; Livny *et al.*, 2007; Ringgaard *et al.*, 2011; Yamaichi & Niki, 2000). Chromosomal ParA proteins belong to the subgroup of P-loop ATPases with a deviant Walker A motif (Koonin, 1993; Motallebi-Veshareh *et al.*, 1990) without N-terminal DNA-binding domains (Fig. 1). Their partners, DNA-binding proteins with an HTH motif in the central part, belong to the highly conserved family of ParB proteins. Chromosomally encoded ParA and ParB homologues can mimic their plasmid counterparts in stabilizing otherwise unstable plasmids (Bartosik *et al.*, 2004; Lin & Grossman, 1998; Yamaichi & Niki, 2000). The importance of chromosomal Par systems in condensation and segregation of *oriC* domains of newly replicated chromosomes prior to cell division has been confirmed (Bartosik *et al.*, 2009; Figge *et al.*, 2003; Fogel & Waldor, 2006; Gruber & Errington, 2009; Lasocki *et al.*, 2007; Ogura *et al.*, 2003; Ptacin *et al.*, 2010; Schofield *et al.*, 2010; Sullivan *et al.*, 2009; Umbarger *et al.*, 2011).

Apart from the main function in the separation of *oriC* domains during chromosome segregation, ParA–ParB proteins participate in the control of replication initiation, cell division, growth and motility (Mierzejewska & Jagura-Burdzy, 2012). These additional roles are species-dependent and seem to depend on their ability to interact with other proteins (Bowman *et al.*, 2008; Gruber & Errington, 2009; Ptacin *et al.*, 2010; Ringgaard *et al.*, 2011; Sullivan *et al.*, 2009; Yamaichi *et al.*, 2012).

The object of our investigations was the *par* system of *Pseudomonas aeruginosa* consisting of ParA, ParB and 10 potential *parS* sites, most of them localized in close proximity to the *oriC* region (Bartosik *et al.*, 2004). ParB creates one to four regularly distributed foci in *P. aeruginosa* cells, which co-localize with the nucleoid and undergo dynamic changes (Bartosik *et al.*, 2009). When in excess, ParB is able to silence the expression of genes placed on the test plasmid near *parS*. Overproduction of ParA and ParB in *P. aeruginosa* causes growth inhibition and defects in chromosome segregation (Bartosik *et al.*, 2004; Lasocki *et al.*, 2007). The *parA* and *parB* genes are not essential for *P. aeruginosa* viability, but the chromosomal *par* mutants show defects in growth, chromosome partitioning and motility. Both proteins interact with each other, forming complexes that are protected from proteolytic digestion (Bartosik *et al.*, 2009; Lasocki *et al.*, 2007). Molecular characterization of *P. aeruginosa* ParB revealed its domain structure (Bartosik, *et al.*, 2004, 2009). The central part of ParB is involved in DNA-binding activity not only through the HTH motif, but also through an additional DNA-binding interface (Kusiak *et al.*, 2011). The C terminus of ParB plays an essential role in self-association (primary dimerization domain) with the vital role of hydrophobic residues at the C terminus of the protein (Bartosik *et al.*, 2004; Mierzejewska *et al.*, 2012), whereas the N-terminal part of ParB is involved in oligomerization of the protein (Kusiak *et al.*, 2011).

Previous analysis of *P. aeruginosa* ParA revealed that this protein has the ability to both self-associate and interact with ParB (Bartosik *et al.*, 2004). This study was aimed at the dissection of ParA from *P. aeruginosa* and identification of the dimerization interface as well as the domains involved in interactions with the ParB partner.

## Methods

### 

#### Bacterial and yeast strains, and growth conditions.

The *Escherichia coli* strains used were: DH5α [F*^−^* (ϕ*80dlacZ*Δ*M15*) *recA1 endA1 gyrA96 thi-1 hsdR17*(r_k_^−^m_k_^+^) *supE44 relA1 deoR* Δ*(lacZYA–argF)U196*], BTH101 [F^−^ *cya-99* *araD139* *galE15* *galK16* *rpsL1* (Str^r^) *hsdR2* *mcrA1* *mcrB1*] (Karimova *et al.*, 1998), BL21 [F^−^ *ompT hsdS*_B_ (r_B_^−^m_B_^−^) *gal dcm* (λ DE3)] (Novagen) and S17-1 [*recA pro hsdR* RP4-2-Tc : : Mu-Km : : Tn*7*] (Simon *et al.*, 1986). *P. aeruginosa* PAO1161 (*leu^−^* r^−^m^+^) was kindly provided by B. M. Holloway (Monash University, Australia). PAO1161 Rif^R^ derivative was used as a recipient strain in conjugation. *Saccharomyces cerevisiae* strain L40 [MATa *trp1 leu2 his3 ade2* LYS : : *lexA*–HIS3 URA3 : : *lexA*–*lacZ*] was provided by Clontech.

Bacteria were grown in L-broth (Kahn *et al.*, 1979) at 37 °C. Some experiments were performed in M9 minimal medium with glucose (Sambrook *et al.*, 1989) supplemented with leucine (132 mM) for propagation of PAO1161 derivative strains. L-agar (L-broth with 1.5 % w/v agar) was supplemented with antibiotics at appropriate concentrations. For *E. coli* strains, benzyl penicillin sodium salt (150 µg ml^−1^ in liquid media and 300 µg ml^−1^ in agar plates), kanamycin sulfate (50 µg ml^−1^) or chloramphenicol (10 µg ml^−1^) were added. For *P. aeruginosa* strains, carbenicillin (300 µg ml^−1^) and rifampicin (300 µg ml^−1^) were applied. L-agar used for blue/white screening contained 0.1 mM IPTG and 40 µg X-Gal ml^−1^. MacConkey agar was supplemented with 1 % maltose, antibiotics and 0.1 mM IPTG.

#### Plasmid DNA isolation, analysis, DNA amplification and manipulation.

Plasmid DNA was isolated and manipulated by standard procedures (Sambrook *et al.*, 1989). Standard PCRs (Mullis *et al.*, 1986) were performed with the appropriate pairs of primers listed in Table S1 (available in the online Supplementary Material). *E. coli* competent cell preparation and DNA transformation were performed according to standard protocols (Sambrook *et al.*, 1989). The fidelity of PCR-derived clones was checked by DNA sequencing (DNA Sequencing and Oligonucleotide Synthesis Laboratory, Institute of Biochemistry and Biophysics, Polish Academy of Sciences, Warsaw, Poland). Plasmids used in this study are listed in Tables 1 and 2. Construction of modified *parA* alleles is described in detail in the Supplementary Materials and Methods. Requests for strains and plasmids constructed in this work should be addressed to the corresponding author.

**Table 1.** Plasmids used in this work

Plasmid	Relevant features	Reference/source
pAKE600	*ori*_MB1_, *oriT*_RK2_, Ap^R^, *sacB*	El-Sayed *et al.* (2001)
pAMB9.37	pBBRMCS-1 expression vector, *lacI^q^ tacp*	Ludwiczak *et al.* (2013)
pBBR1MCS-1	IncA/C broad-host-range cloning vector, *lacZ*α–MCS, *mob*, T7p, T3p, Cm^R^	Kovach *et al.* (1994)
pBGS18	*ori*_MB1_, Km^R^, cloning vector	Spratt *et al.* (1986)
pBTM116	*ori*_MB1_, 2μ, Ap^R^, *trp1*, shuttle vector *lexA*_BD_	Clontech
pET28a(+)	*ori*_MB1_, Km^R^, T7p, *lacO*, His_6_-tag, T7 tag, expression vector	Novagen
pET28mod	*ori*_MB1_, Km^R^, T7p, *lacO*, His_6_-tag, modified to remove T7 tag	Lukaszewicz *et al.* (2002)
pGAD424	*ori*_MB1_, 2μ, Ap^R^, *leu2*, shuttle vector *gal4*_AD_	Clontech
pKLB1.4	pGAD424 with *gal4*_AD_–*parA* translational fusion	Bartosik *et al.* (2004)
pKLB1.6	pBTM116 with *lexA*_DB_–*parA* translational fusion	Bartosik *et al.* (2004)
pKLB2.4	pGAD424 with *gal4*_AD_–*parB* translational fusion	Bartosik *et al.* (2004)
pKLB2.6	pBTM116 with *lexA*_DB_–*parB* translational fusion	Bartosik *et al.* (2004)
pKLB28	pET28mod with *T7p*–*parB* transcriptional fusion	Bartosik *et al.* (2004)
pKLB60.1	pAKE600 derivative lacking *Bam*HI site	Lasocki *et al.* (2007)
pKLB60.2	pAKE600 derivative with *parA*	Lasocki *et al.* (2007)
pKLB8.1	pET28mod with *T7p*–*parA* transcriptional fusion	Lasocki *et al.* (2007)
pKNT25	*ori*_p15_, Km^R^, *lacp*–*MCS*–*cyaT25*	Karimova *et al.* (1998)
pKT25	*ori*_p15_, Km^R^, *lacp*–*cyaT25*–*MCS*	Karimova *et al.* (1998)
pKT25-zip	pKT25 with *lacp*–*cyaT25*–GCN4 leucine zipper fragment	Karimova *et al.* (1998)
pLKB2	pKT25 with modified MCS	L. Kusiak*
pLKB220	pLKB2 with translationally fused *cyaT25*–*parA*	L. Kusiak
pLKB233	pLKB2 with translationally fused *cyaT25*–*parB*	L. Kusiak
pLKB4	pUT18C derivative with modified MCS	L. Kusiak
pMKB5.1	pLKB4 with *cyaT18*–*parA* translational fusion	M. Kusiak*
pMKB5.2	pLKB4 with *cyaT18*–*parB* translational fusion	M. Kusiak
pMKB5.3	pUT18 with *parA*–*cyaT18* translational fusion	M. Kusiak
pMKB5.4	pUT18 with *parB*–*cyaT18* translational fusion	M. Kusiak
pMKB6.1	pKNT25 with *parA*–*cyaT25* translational fusion	M. Kusiak
pMKB6.2	pKNT25 with *parB*–*cyaT25* translational fusion	M. Kusiak
pUC18	*ori*_MB1_, Ap^R^, cloning vector	Yanisch-Perron *et al.* (1985)
pUT18	*ori*_ColE1_, Ap^R^, *lacp*–*MCS*–*cyaT18*	Karimova *et al.* (1998)
pUT18C	*ori*_ColE1_, Ap^R^, *lacp*–*cyaT18*–*MCS*	Karimova *et al.* (1998)
pUT18C-zip	pUT18C with *lacp*–*cyaT18*–GCN4 leucine zipper fragment	Karimova *et al.* (1998)

MCS, multiple cloning site.

* Institute of Biochemistry and Biophysics, Polish Academy of Sciences.

**Table 2.** Plasmids constructed during this work.

Plasmid	Relevant features
**pAKE600 derivatives**	
pKGB6.67	*parA67* inserted as *Eco*RI/*Sal*I fragment
pKLB60.4	PCR-amplified *parA1*–*47* with the use of primers 1 and 6, inserted as *Eco*RI/*Hin*dIII fragment
pKLB60.5	*parA*Δ*48*–*59*, PCR-amplified fragment (primers 8 and 2) encoding *parA60*–*262* cloned as *Hin*dIII/*Sal*I fragment into pKLB60.4
pKLB60.6	PCR-amplified *parA1*–*76* with the use of primers 1 and 7, inserted as *Eco*RI/*Hin*dIII fragment
pKLB60.7	*parA*Δ*77*–*85*, PCR-amplified fragment (primers 9 and 2) encoding *parA86*–*262* cloned as *Hin*dIII/*Sal*I fragment into pKLB60.6
pKLB60.8*	*parA84* inserted as *Eco*RI/*Sal*I fragment
**pUC18 derivatives**	
pGMB11*	*parA74* inserted as *Eco*RI/*Sal*I fragment
pGMB12*	*parA75* inserted as *Eco*RI/*Sal*I fragment
pGMB13*	*parA76* inserted as *Eco*RI/*Sal*I fragment
pGMB14*	*parA78* inserted as *Eco*RI/*Sal*I fragment
pGMB15*	*parA79* inserted as *Eco*RI/*Sal*I fragment
**pBGS18 derivatives**	
pGMB33*	*parA* inserted as *Eco*RI/*Sal*I fragment with internal *Sac*I site without amino acid change
**Bacterial two-hybrid vectors**	
**pKGB4**	**pUT18 derivative** with modified MCS to facilitate ORF cloning as *Eco*RI/*Kpn*I or *Eco*RI/*Sac*I fragments in-frame with the N terminus of CyaT18
pKGB4.14	pKGB4 with *parA67*–*cyaT18* translational fusion
pKGB4.15	pKGB4 with *parA68*–*cyaT18* translational fusion
pKGB4.16	pKGB4 with *parA70*–*cyaT18* translational fusion
pKGB4.17	pKGB4 with *parA72*–*cyaT18* translational fusion
pKGB4.18	pKGB4 with *parA74*–*cyaT18* translational fusion
pKGB4.19	pKGB4 with *parA75*–*cyaT18* translational fusion
pKGB4.20	pKGB4 with *parA76*–*cyaT18* translational fusion
pKGB4.21	pKGB4 with *parA78*–*cyaT18* translational fusion
pKGB4.22	pKGB4 with *parA79*–*cyaT18* translational fusion
pKGB4.24	pKGB4 with *parA82*–*cyaT18* translational fusion
pKGB4.25	pKGB4 with *parA83*–*cyaT18* translational fusion
pKGB4.26	pKGB4 with *parA84a*†–*cyaT18* translational fusion
pKGB4.27	pKGB4 with *parA84*–*cyaT18* translational fusion
pKGB4.28	pKGB4 with *parA85*–*cyaT18* translational fusion
**pKGB5**	**pKNT25 derivative** with modified MCS to facilitate ORF cloning as *Eco*RI/*Kpn*I or *Eco*RI/*Sac*I fragments in-frame with the N terminus of CyaT25
pKGB5.14	pKGB5 with *parA67*–*cyaT25* translational fusion
pKGB5.15	pKGB5 with *parA68*–*cyaT25* translational fusion
pKGB5.16	pKGB5 with *parA70*–*cyaT25* translational fusion
pKGB5.17	pKGB5 with *parA72*–*cyaT25* translational fusion
pKGB5.18	pKGB5 with *parA74*–*cyaT25* translational fusion
pKGB5.19	pKGB5 with *parA75*–*cyaT25* translational fusion
pKGB5.20	pKGB5 with *parA76*–*cyaT25* translational fusion
pKGB5.21	pKGB5 with *parA78*–*cyaT25* translational fusion
pKGB5.22	pKGB5 with *parA79*–*cyaT25* translational fusion
pKGB5.24	pKGB5 with *parA82*–*cyaT25* translational fusion
pKGB5.25	pKGB5 with *parA83*–*cyaT25* translational fusion
pKGB5.26	pKGB5 with *parA84a*–*cyaT25* translational fusion
pKGB5.27	pKGB5 with *parA84*–*cyaT25* translational fusion
pKGB5.28	pKGB5 with *parA85*–*cyaT25* translational fusion
**pBBRMCS-1 derivative**	
pABB1.0	pBBRMCS-1 with modified *Eco*RI restriction site within the Cm^R^ cassette (PCR site-directed mutagenesis with primers 26 and 27)
pABB1.2	pAMB9.37 with *tacp*–*parB* transcriptional fusion
pABB84	pAMB9.37 with *tacp*–*parA84a* transcriptional fusion
**pET28a (+) or pET28mod derivatives**	
pABB8.0	*parA* fragment PCR-amplified with primers 3 and 4 and inserted as *Nco*I/*Xho*I
pABB8.67	*parA67* PCR-amplified and inserted as above
pABB8.78	*parA78* PCR-amplified and inserted as above
pABB8.83	*parA83* PCR-amplified and inserted as above
pABB8.84	*parA84a* PCR-amplified and inserted as above
pKLB8.3	*parA40*–*262* inserted as *Eco*RI/*Sal*I fragment
pKLB8.4	*parA1*–*40* inserted as *Eco*RI/*Sal*I fragment
pKLB8.5	*parA1*–*151* inserted as *Eco*RI/*Sal*I fragment
pKLB8.6	*parA152*–*262* inserted as *Eco*RI/*Sal*I fragment
pKLB8.7	*parA40*–*151* inserted as *Eco*RI/*Sal*I fragment
pKLB8.8	*parA1*–*85* inserted as *Eco*RI/*Sal*I fragment
pKLB8.9	*parA86*–*151* inserted as *Eco*RI/*Sal*I fragment
pKLB8.10	*parA*Δ*48*–*59* inserted as *Eco*RI/*Sal*I fragment
pKLB8.11	*parA*Δ*77*–*85* inserted as *Eco*RI/*Sal*I fragment
**Yeast two-hybrid vectors**	
pABB482*	pGMB57 with *parA82* inserted as *Eco*RI/*Sal*I fragment
pABB483*	pGMB57 with *parA83* inserted as *Eco*RI/*Sal*I fragment
pABB484*	pGMB57 with *parA84a* inserted as *Eco*RI/*Sal*I fragment
pABB485*	pGMB57 with *parA85* inserted as *Eco*RI/*Sal*I fragment
pGMB21*	pGAD424 with *parA67* inserted as *Eco*RI/*Sal*I fragment
pGMB22*	pGAD424 with *parA68* inserted as *Eco*RI/*Sal*I fragment
pGMB24*	pGAD424 with *parA70* inserted as *Eco*RI/*Sal*I fragment
pGMB25*	pGAD424 with *parA72* inserted as *Eco*RI/*Sal*I fragment
pGMB57	pGAD424 with *gal4*_AD_–*parA40*–*262* fragment with internal *Sac*I site inserted as *Bam*HI/*Sal*I fragment
pKLB4.5	pGAD424 with *gal4*_AD_–*parA1*–*151* inserted as *Eco*RI/*Sal*I fragment
pKLB4.8	pKLB4.5 with *gal4*_AD_–*parA1*–*85* with *Sal*I oligo with stop codon inserted in *Nae*I site
pKLB4.9	pKLB4.5 with *gal4*_AD_–*parA86–151* with *Eco*RI oligo with ATG codon inserted in *Nae*I site, *Eco*RI fragment deleted
pKLB6.3	pBTM116 with *lexA*_DB_–*parA40*–*262* inserted as *Bam*HI/*Sal*I fragment
pKLB6.4	pBTM116 with *lexA*_DB_–*parA1*–*40* inserted as *Eco*RI/*Sal*I fragment
pKLB6.5	pBTM116 with *lexA*_DB_–*parA1*–*151* inserted as *Eco*RI/*Sal*I fragment
pKLB6.6	pBTM116 with *lexA*_DB_–*parA152–262* inserted as *Eco*RI/*Sal*I fragment
pKLB6.7	pBTM116 with *lexA*_DB_–*parA40*–*151* inserted as *Eco*RI/*Sal*I fragment
pKLB6.8	pBTM116 with *lexA*_DB_–*parA1*–*85* inserted as *Eco*RI/*Sal*I fragment
pKLB6.9	pBTM116 with *lexA*_DB_–*parA86–151* inserted as *Eco*RI/*Sal*I fragment
pKLB6.10	pBTM116 with *lexA*_DB_–*parA*Δ*48*–*59* inserted as *Eco*RI/*Sal*I fragment
pKLB6.11	pBTM116 with *lexA*_DB_–*parA*Δ*77*–*85* inserted as *Eco*RI/*Sal*I fragment

* Construction of these plasmids is described in detail in Supplementary Materials and Methods.

† *parA84a* encodes ParAL84A, whereas *parA84* encodes ParAL84K.

#### Yeast two-hybrid (YTH) system.

The *parA* alleles were cloned in two vectors, pGAD424 and pBTM116, to form translational fusions with C termini of GAL4 and LexA, respectively. *S. cerevisiae* strain L40 was transformed with pGAD424 and pBTM116 *parA* mutant derivatives in various combinations with adequate plasmids encoding either WT ParA or WT ParB. Yeast strain transformation and LacZ activity assay were performed as described previously (Bartosik *et al.*, 2004). Colonies of double transformants were transferred on nitrocellulose filters, immersed in liquid nitrogen to lyse and treated with X-Gal solution as a substrate.

#### Bacterial two-hybrid system.

A bacterial adenylate cyclase two-hybrid (BACTH) system was used to analyse *in vivo* interactions between ParA single-amino-acid substitution derivatives and WT ParA or ParB (Karimova *et al.*, 1998, 2000). The multiple cloning site (MCS) sequences in the original BACTH system vectors were modified according to requirements to construct two pairs of vectors: pLKB2 and pLKB4 or pKGB4 and pKGB5 (Table 2).

For protein–protein interactions in the BACTH assay, pairs of appropriate vectors were used to co-transform *E. coli* BTH101 *cyaA^−^* cells. The co-transformants were selected onto MacConkey agar supplemented with 1 % maltose, 0.1 mM IPTG, kanamycin and penicillin, and grown at 30 °C for 48 h. The β-galactosidase activity assays were performed according to Miller (1972).

#### His_6_-tagged proteins purification.

The *E. coli* BL21(DE3) strain was transformed with pET28 derivatives encoding ParA variants His_6_-tagged at C ends. Overproduction of ParA-His_6_ or its mutant forms was carried out overnight in M9 medium with glucose supplemented with kanamycin (50 µg ml^−1^) and 0.1 mM IPTG. Tris buffer (10 mM Tris/HCl, pH 8, 1 M NaCl, 0.1 mM EDTA and 5 % glycerol) was used during the purification procedure on Protino Ni-TED 1000 columns (Macherey-Nagel). Purified proteins were stored in small portions at −80 °C prior to further analysis.

His_6_-ParB purification was carried out as described previously (Bartosik *et al.*, 2004); however, Tris buffer (as for ParA) was used instead of phosphate buffer.

#### ATPase activity assay.

ATPase activity of purified ParA-His_6_ and its mutant derivatives was determined using the Malachite Green Phosphate Assay kit (BioAssay Systems). The purified protein (100 pmol) was incubated in 50 mM Tris/HCl, pH 8, 150 mM NaCl, 10 mM MgCl_2_, 5 % glycerol, 1 mM ATP and 2 mg BSA ml^−1^ in a final volume of 50 µl. The amount of released phosphate was assayed at 30 min intervals (up to 2 h) and calculated according to the phosphate standard curve prepared under the same conditions. At least 10 measurements in three independent experiments were performed for each ParA derivative using a 96-well microplate (Greiner Bio-One) in a Bio Tek plate reader at 630 nm.

#### Co-immunoprecipitation after *in vivo* protein cross-linking with formaldehyde.

*E. coli* BL21(DE3) strain was transformed with pET28 derivatives carrying different *parA* alleles and pABB1.2 carrying a *tacp*–*parB* fusion. Formaldehyde was added to a final concentration of 1 % to the exponentially growing cultures induced for 4 h by 0.5 mM IPTG. The cells were incubated for 30 min at room temperature, pelleted and washed twice in 10 ml PBS buffer (15 mM KCl, 150 mM NaCl and 10 mM NaPi, pH 7.4). The pellet was resuspended in 100 µl lysis buffer (10 mM Tris/HCl, pH 8, 20 % sucrose and 40 mM EDTA) with 1 mg lysozyme ml^−1^ and incubated on ice for 30 min. Then 100 µl 2× IP buffer (1.5 M Tris/HCl, pH 7, 300 mM NaCl and 0.2 % Triton X-100) with 1 mM PMSF was added. After 10 min on ice, the cell suspensions were sonicated and cleared by centrifugation. An aliquot of 100 µl anti-ParB antibodies was added to the soluble fraction and incubated overnight on an orbital shaker at 4 °C. Then 50 µl Protein A–Sepharose (Amersham Biosciences) was added to each sample and treated according to the protocol. Western blotting with anti-His_6_-tag antibodies (1 : 3000 dilution; Pierce) was carried out after protein separation by SDS-PAGE and transfer onto a nitrocellulose membrane.

#### Introduction of mutant alleles into the PAO1161 backbone by homologous recombination.

The *parA* mutant alleles were cloned as *Eco*RI/*Sal*I fragments into pKLB60.1, a derivative of the suicide vector pAKE600 (El-Sayed *et al.*, 2001). The constructed plasmids were transformed into *E. coli* S17-1 and then mobilized into *P. aeruginosa* PAO1161 Rif^R^. The allele exchange procedure was performed as described previously (Lasocki *et al.*, 2007). The allele exchange was verified by sequencing of PCR fragments amplified on the mutant chromosomal DNA as the template.

#### Fluorescence microscopy.

DAPI staining and immunofluorescence microscopy were carried out as described previously (Bartosik *et al.*, 2004; Bignell *et al.*, 1999). Cells were analysed using a Nikon Eclipse EC 800 microscope. The images were collected and analysed in Lucia software, and prepared for publication using Adobe Photoshop CS4.

#### Motility assays.

Motility assays were performed as described previously (Rashid & Kornberg, 2000). All sets of plates were standardized by using the same medium volume.

#### Modelling.

A structural model of the monomeric ParA of *P. aeruginosa* was obtained using sybyl-x 2.0 (Tripos) on the basis of ParA of *P. aeruginosa* and Soj of *Thermus thermophilus* alignment as well as the Soj crystal structure (Protein Data Bank ID: 1WCV; Leonard *et al.*, 2005). To check if introduced amino acid substitutions significantly influenced ParA structure, the models of WT ParA and its derivatives were subjected to energy minimization using the AmberFF99 force field as implemented in sybyl-x 2.0.

## Results

### *In vivo* deletion mapping of the ParA domains involved in dimerization and interactions with ParB

**Fig. 2.**
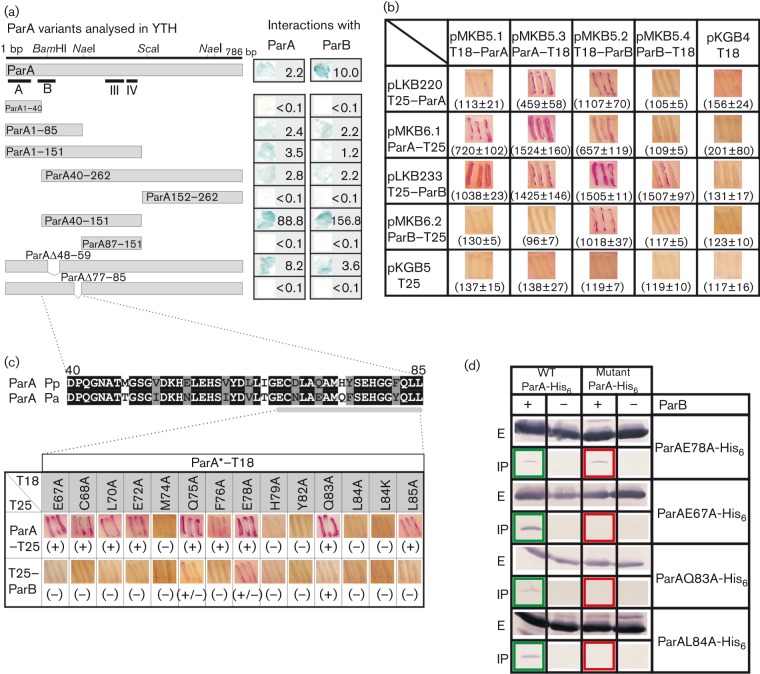
Protein–protein interactions of ParA mutants. (a) ParA deletion mutant analysis in the YTH system. *S. cerevisiae* strain L40 was transformed with the appropriate pairs of pGAD424 and pBTM116 derivatives carrying *parA*, *parB* and different *parA* deletion alleles. The interactions between hybrid proteins were visualized by the plate test and β-galactosidase activity assays in liquid cultures. The mean values of LacZ activities from at least three independent experiments are shown. (b) BACTH system analysis of *P. aeruginosa* ParA and ParB interactions. *E. coli* BTH101 *cyaA*^−^ was transformed with the pairs of BACTH vectors. As the control, double transformants of the plasmids encoding hybrid proteins and empty vectors were included. Data in parentheses represent the mean±sd β-galactosidase values from at least three experiments. (c) BACTH analysis of ParA substitution derivatives. (Upper) Comparison of ParA region D40–L85 from *P. aeruginosa* (Pa) and *P. putida* (Pp), with identical (black background) and similar (grey background) residues indicated. The ParA region analysed by alanine scanning is enlarged. (Lower) Summary of BACTH results between mutated ParAs linked to CyaAT18 and ParA–CyaAT25 or CyaAT25–ParB. ParA* represents ParA derivatives with amino acid substitutions. (+), Interactions detected; (+/−), weak interactions; (–), no interactions. (d) Co-immunoprecipitation of ParB with ParA derivatives. The extracts of strains producing His_6_-tagged ParA derivatives with/without WT ParB (‘+’ and ‘–’, respectively) were treated with anti-ParB antibodies. The ParA derivatives in immunoprecipitated pellets (‘IP’ panels) were detected with anti-His_6_ antibodies. ‘E’ panels show ParA protein levels in the initial extracts. In each set of experiments WT ParA was co-immunoprecipitated with ParB as the control.

It has been shown previously using the YTH system that ParA of *P. aeruginosa* dimerizes and is able to interact with ParB (Bartosik *et al.*, 2004). To establish which part of ParA is responsible for dimerization and interactions with ParB, a set of *parA* deletion mutants (Fig. 2a) was constructed (Supplementary Materials and Methods) and tested in the YTH system. The *parA* derivatives were used as bait or prey by translational linking with the activation domain of GAL4 or the DNA-binding domain of LexA (pGAD424 and pBTM116, respectively), and tested for interactions with hybrid *parA* and *parB* cloned into complementary vectors. The *S. cerevisiae* L40 strain was transformed with the appropriate pairs of plasmids. The expression of the *lexA*–*lacZ* fusion, activated by interactions between hybrid proteins, was monitored in double transformants by plate tests and β-galactosidase activity assays in the liquid cultures. Regardless of ParA derivatives being the bait or prey, the results were the same so only the results for one combination of hybrid proteins are demonstrated in Fig. 2(a) as scanned nitrocellulose filters, the corresponding enzyme activities assayed in the liquid cultures are shown.

Both truncated proteins, ParA40–262 and ParA1–151, self-associated in the YTH system, indicating that neither the N-terminal 39 aa nor the C-terminal 111 aa were important for ParA dimerization *in vivo* (Fig. 2a). Interestingly, when both the N and C terminus were removed (ParA40–151), the dimerization ability (measured by β-galactosidase activity) was significantly stronger than that detected for the single deletions. ParA1–85 was also able to self-associate in the YTH system and, as ParA1–40 and ParA87–151 could not, it was concluded that the short region between D40 and L85 played an important role in ParA self-association.

The same truncated forms of ParA that gave positive results in the dimerization test demonstrated interactions with ParB (Fig. 2a). ParA40–151 interacted with ParB significantly more strongly than any other modified form of ParA (similarly to the enhanced effect of association with ParA). This suggested that the 46 aa segment located between D40 and L85 was involved in both ParA dimerization and ParA–ParB interactions, and the removal of the N and C termini unmasked the interaction domain of ParA.

The ParA alignment (Fig. 1) showed that the D40–L85 segment encompassed two highly conserved blocks and two short sequences quite diverse among various bacterial strains, but very similar in two *Pseudomonas* species, *P. aeruginosa* and *Pseudomonas putida* (Fig. 2c). As such regions of variability between homologous proteins from different species may reflect important species specificity of the partitioning components, two short internal deletion alleles encoding ParAΔ48–59 and ParAΔ77–85, lacking the amino acid residues from the *Pseudomonas* ‘specific’ regions, were constructed (Supplementary Materials and Methods). The YTH assay revealed that the deletion of 12 aa in ParAΔ48–59 did not eliminate self-association and interactions with ParB, whereas the ParAΔ77–85 derivative was not able to dimerize or interact with ParB (Fig. 2a).

The low level of hybrid protein production in the yeast strains did not allow their detection by Western blotting, so the possibility that lack of interactions resulted from the instability of some derivatives in the yeast strains could not be excluded.

### Fine mapping of dimerization and ParB interaction determinants in ParA

As the YTH analysis indicated quite weak ParA self-association and ParA–ParB interactions, it was decided to shift to the BACTH system (Karimova *et al.*, 1998, 2000) to potentially increase the sensitivity of detection of interactions between Par proteins and possibly monitor the stability of hybrid proteins.

The *parA* and *parB* alleles were cloned into two pairs of BACTH vectors, derivatives of pairs pUT18C/pKT25 and pUT18/pKNT25, facilitating the linkage of Par proteins with two adenylate cyclase (CyaA) fragments (T18 and T25) of *Bordetella pertussis*, either at the N or C-terminus of tested proteins (Tables 1 and 2). Reconstruction of adenylate cyclase activity due to the interactions between proteins fused to the CyaA fragments led to the production of cAMP, which bound to the activator CAP and turned on the expression of sugar catabolism genes (e.g. *lac* or *mal* operon). The plate test for maltose fermentation and β-galactosidase assays in the liquid cultures of double transformants of *E. coli* BTH101 *cyaA^−^* strain demonstrated that the strongest ParB–ParB interactions occurred between ParB linked to the CyaA fragment by its N terminus (CyaA–ParB fusions), whereas the strongest dimerization of ParA was detected when ParA was linked to the CyaA fragment by its C terminus (ParA–CyaA fusions) (Fig. 2b).

Interactions between ParA and ParB were undetectable when ParB–CyaA was tested, suggesting that the C terminus of ParB is important for both homo- and heterologous interactions. The CyaA–ParB fusions demonstrated strong interactions with ParA regardless of which ParA end was linked to the CyaA fragment.

The YTH analysis indicated that region 59–84 in ParA is important for dimerization and interactions with ParB. Part of this region (60–64) is highly conserved among various chromosomal ParA homologues (Fig. 1). We decided to modify residues that were variable in ParAs from different classes, but similar in the *Pseudomonas* genus.

Thirteen *parA* point mutant alleles with alanine substitutions in the region E67–L85 (Fig. 2c) were constructed (Supplementary Materials and Methods). The previously constructed allele *parAL84K* was also included in the analysis. All mutant alleles were cloned into two BACTH system vectors, pKGB4 and pKGB5, facilitating their translational fusion via C-termini to two CyaA fragments, T18 and T25, respectively. Western analysis of extracts from DH5α cells carrying pKGB4 derivatives showed no differences in the stability of the hybrid proteins (Fig. S1). The BTH101 *cyaA^−^* strain was transformed with new constructs in pairs with vectors carrying *parA*–*cyaA* and *cyaA*–*parB* fusions to check the ability of mutant derivatives to dimerize and interact with ParB. The data shown in Fig. 2(c) confirmed that the E67–L85 region was very important for protein–protein interactions and divided the ParA substitution mutants into three categories. The first category consisted of two variants (ParAQ75A and E78A) that could dimerize similar to WT ParA and had slightly decreased ability to interact with ParB. The second category encompassed five mutants (ParAM74A, H79A, Y82A, L84A and L84K) that were defective in dimerization and interactions with ParB. The third category included seven mutants (ParAE67A, C68A, L70A, E72A, F76A, Q83A and L85A) that were capable of self-interactions, but did not interact with ParB, suggesting that the interface involved in ParA dimerization is not identical to the interface involved in interactions with ParB. As none of the ParA mutants defective in dimerization interacted with ParB, it was concluded that ParA dimerization was vital for association with ParB.

### Verification of ParA–ParB interactions

Four *parA* alleles were chosen for further analysis: *parAE67A* and *parAQ83A* encoded ParAs that could dimerize, but did not interact with ParB, *parAE78A* encoded a product that was only slightly impaired in its ability to interact with ParB, and *parAL84A* encoded a product that showed neither dimerization nor interactions with ParB.

An immunoprecipitation assay was used to check the interactions between Par proteins. *E. coli* BL21(DE3) cells were transformed with pET28 derivatives overproducing the four mutant ParAs His_6_-tagged at their C-termini and with a pBBR1-MCS1 derivative carrying a *tacp*–*parB* transcriptional fusion (pABB1.2). In each set of immunoprecipitation experiments, strain BL21(DE3) (pABB8.0) (pABB1.2), overproducing WT ParA-His_6_ and WT ParB, was included as the positive control. After protein overproduction, the formaldehyde cross-linked complexes were immunoprecipitated with polyclonal anti-ParB antibodies, and anti-His_6_-tag antibodies were used to detect the presence of ParA in the initial extracts and immunoprecipitation pellets (Fig. 2d). The results showed that ParB formed complexes only with WT ParA and ParAE78A. ParAE67A, ParAQ83A and ParAL84A were not co-precipitated with ParB, confirming that the introduced amino acid substitutions significantly impaired the interactions between partners.

### ATPase activity of WT ParA and its mutant derivatives.

Previous studies on various ParA homologues demonstrated their weak ATPase activities (Batt *et al.*, 2009; Barillà & Hayes, 2003; Bouet & Funnell, 1999; Bouet *et al.*, 2007; Leipe *et al.*, 2002; Leonard *et al.*, 2005; Lutkenhaus & Sundaramoorthy, 2003; Pratto *et al.*, 2008).

**Fig. 3.**
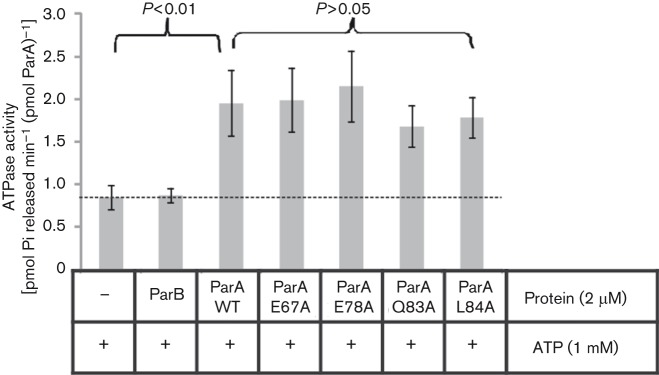
ATPase activities of ParA mutant derivatives. The C-terminally His_6_-tagged purified ParA derivatives (2 µM) were incubated with ATP and released inorganic phosphate (Pi) was detected spectrophotometrically. The control reactions (no protein added or His_6_-ParB alone) were included. Data represent the mean±sd values from at least 10 experiments. Statistical analysis revealed significant differences between WT ParA and two control samples (*P*<0.01; *t*-test), but not between different ParA variants (*P*>0.05; *t*-test).

The *parA* gene was cloned under the control of T7p into pET28a(+), expressed in *E. coli* BL21(DE3) and C-terminally His_6_-tagged ParA (ParA-His_6_) was purified (Fig. S2). The ATPase activity of ParA-His_6_ was assayed by the spectrophotometric detection of released inorganic phosphate (Pi). ParA-His_6_ demonstrated ATPase activity (Fig. 3) of ~1.2 pmol Pi released min^−1^ (pmol ParA)^−1^.

Four ParA variants (tested by immunoprecipitation) were purified by affinity chromatography after overproduction in *E. coli* BL21(DE3) transformed with pET28a(+) derivatives carrying these *parA* alleles. All analysed ParA derivatives, among them dimerization-deficient ParAL84A, demonstrated ATPase activities similar to WT ParA (Fig. 3). This suggested that the amino acid substitutions did not drastically change the protein folding responsible for enzymic activity (see also Fig. 5b and Discussion).

### Introduction of *parA67* and *parA84* mutations into the chromosome of PAO1161

The alleles *parA84* and *parA67* were chosen to be introduced into the *P. aeruginosa* PAO1161 genome as representatives of two different classes of *parA* mutants. ParAL84K was unable to dimerize and interact with ParB, whereas ParAE67A was defective in interactions with ParB, but capable of self-association. The mutant alleles were cloned into the suicide vector pAKE600 (El-Sayed *et al.*, 2001) and then introduced into the PAO1161 chromosome via homologous recombination (allele exchange).

**Fig. 4.**
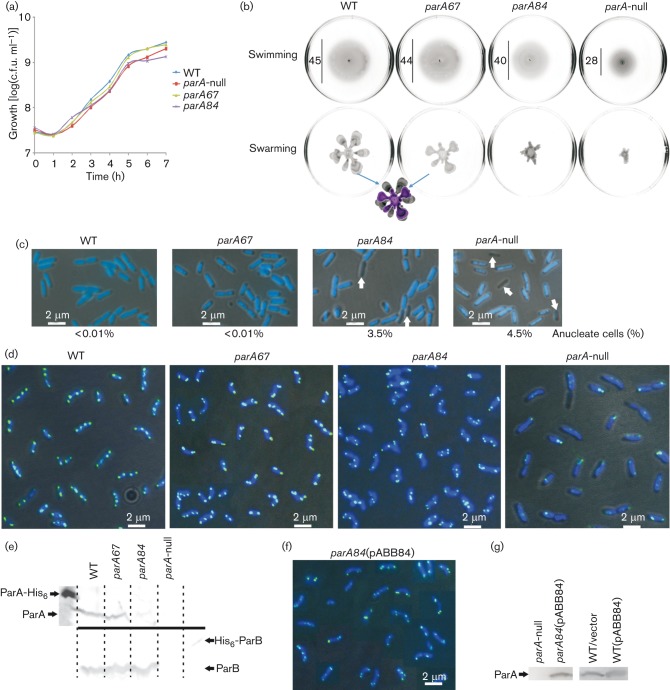
Effect of *parA84* and *parA67* mutations on growth, motility, nucleoid segregation and ParB localization in *P. aeruginosa*. Data for the PAO1161 WT strain and PAO1161 *parA*-null mutant are included for comparison. (a) Growth of WT, *parA*-null, *parA67* and *parA84* strains (L-broth, 37 °C) presented as log(c.f.u. ml^−1^). (b) Swimming and swarming assays. Representative images are shown. The diameters of swimming zones are indicated (in mm). The swarming zones of WT PAO1161 and *parA67* are overlaid for comparison. (c) Anucleate cell formation. Representative images of cells from the exponential growth phase (L-broth, 37 °C) are shown. Anucleate cells are indicated by arrows. (d) Subcellular localization of ParB. Representative images of cells from the exponential growth phase (L-broth, 37 °C) are shown. The dark background in the merged micrographs is a phase-contrast image, the dark blue is the DAPI-stained chromosome and the green/light blue is the FITC-stained ParB. (e) Par protein levels in the mutant strains. ParA (top, anti-ParA antibodies) and ParB (bottom, anti-ParB antibodies) detected in 10^9^ cells from the exponentially growing cultures of analysed strains by Western blotting. ParA-His_6_ (200 ng) and His_6_-ParB (30 ng) were used as the respective controls. (f) Fluorescence detection of ParB in the merodiploid strain PAO1161 *parA84* (pABB84). Description as in (d). (g) ParA levels in the merodiploid strains. Western blotting with anti-ParA antibodies was used to visualize ParA proteins in extracts from 10^9^ cells. Bar, 2 *μ*m.

Growth experiments performed in L-broth (Fig. 4a) at 37 °C showed a slower growth rate of the PAO1161 *parA84* mutant strain (mean division time 45 min) as compared with the parental PAO1161 strain (mean division time 36 min), the phenotype that was observed previously for the *parA*-null mutant (Lasocki *et al.*, 2007). The mutant PAO1161 *parA67* showed ~10 % reduction in growth rate (mean division time 39 min).

The PAO1161 *parA84* and *parA67* mutants were also tested for motility properties (Fig. 4b). The PAO1161 *parA84* mutant showed defects in swarming and swimming, although not as strong as the *parA-*null mutant used as the control strain (Lasocki *et al.*, 2007). The PAO1161 *parA67* mutant was not impaired in swimming and only slightly defective in swarming as compared with the WT strain.

Cells collected from the exponential phase of culture growth were stained with DAPI, and the presence of anucleate cells was monitored in the newly constructed *parA* mutants and control strains, PAO1161 and PAO1161 *parA*-null mutant. Visible defects in chromosome segregation were observed for the PAO1161 *parA84* mutant strain (Fig. 4c); a significant fraction (>3.5 %) of chromosome-less cells or cells with partially segregated chromosomes appeared. A similar proportion of chromosome-less cells was observed for the PAO1161 *parA*-null, whereas parental PAO1161 and mutant PAO1161 *parA67* strains produced <0.01 % visibly defective cells.

As both ParA derivatives encoded by the mutated alleles were impaired in interactions with ParB, it was important to determine the effect of such modifications on ParB distribution in *P. aeruginosa* cells. Immunofluorescence microscopy using anti-ParB antibodies and FITC-conjugated secondary antibodies was used to observe cells from exponential-phase cultures grown on rich medium at 37 °C.

The majority of the actively dividing cells of PAO1161 contained two to four ParB foci distributed symmetrically along the long cell axis, marking the positions of *oriC* domains of the *P. aeruginosa* chromosome (Bartosik *et al.*, 2009), whereas the signals for ParB in the *parA*-null mutant were much weaker, irregularly distributed and often in pairs (Fig. 4d). The PAO1161 *parA84* strain showed perturbations in ParB localization that did not conform to the WT pattern of distribution (Fig. 4d). ParB signals were visibly paired at the poles in 90 % of the cells as compared with 10 % of cells with paired foci in the WT strain. In the case of PAO1161 *parA67*, a similar pattern of non-separated ParB foci was observed although not as frequent as for *parA84* mutant (~40 % of the cells). This indicated that the mutant strains producing ParA impaired in its ability to interact with ParB were defective in proper separation of the *oriC* domains. The stronger mutant phenotype was observed for the ParA derivative impaired not only in interactions with ParB, but also in self-interactions (PAO1161 *parA84*).

The extracts from 10^9^ cells from exponentially growing cultures of WT PAO1161, PAO1161 *parA84* and PAO1161 *parA67* mutants were analysed by Western blotting with anti-ParA antibodies to determine the level of production of ParA variants. Whereas ParAE67A was produced in quantities comparable with the WT ParA in PAO1161 (estimated 400 molecules per cell), the amount of ParAL84K seemed to be at least fivefold lower (Fig. 4e). Both *parA* mutant strains produced similar levels of ParB. To exclude the possibility that the observed mutant phenotypes resulted from insufficient quantities of ParAL84K, the *parA84* allele was cloned into the medium-copy broad-host-range expression vector under *tacp* control to give pABB84. The amount of ParA (with substituted leucine at position 84) produced in the transformant PAO1161 *parA84* (pABB84) was approximately twofold higher than WT ParA in PAO1161 as demonstrated by Western blotting (Fig. 4g). Immunofluorescence experiments with PAO1161 *parA84* (pABB84) cells confirmed that disturbed ParB foci localization/separation was not associated with the ParA level, but resulted from the change in amino acid sequence (Fig. 4f). Additionally, the presence of pABB84 in PAO1161 *parA84* did not reverse other defects, i.e. slower growth rate, motility defects and anucleate cells production (data not shown).

## Discussion

Many studies on Par homologues (plasmid and chromosomally encoded) have demonstrated a role for a dynamic ParA scaffold and the importance of ParA–ParB interactions in bacterial DNA segregation (reviewed by Gerdes *et al.*, 2010; Banigan *et al.*, 2011; Hwang *et al.*, 2013; Lim *et al.*, 2014; Scholefield *et al.*, 2011; Vecchiarelli *et al.*, 2013). In the case of chromosomally encoded Par proteins, their roles in regulation of other cellular processes in a species-specific manner were also demonstrated (Kadoya *et al.*, 2011; Murray & Errington, 2008; Scholefield *et al.*, 2011; Thanbichler & Shapiro, 2006; Yamaichi *et al.*, 2012). Whilst the ParB homologues have been dissected with respect to DNA binding, and dimerization/oligomerization domains, and in many cases the domains of interactions with the ParA homologues identified (Ah-Seng *et al.*, 2009; Barillà *et al.*, 2007; Bartosik *et al.*, 2004; Figge *et al.*, 2003; Kim & Shim, 1999; Leonard *et al.*, 2004; Lukaszewicz *et al.*, 2002; Scholefield *et al.*, 2011; Surtees & Funnell, 1999), less is known about regions of ParA homologues involved in reciprocal interactions with the cognate partners (Jakimowicz *et al.*, 2007; Leonard *et al.*, 2005; Ravin *et al.*, 2003; Scholefield *et al.*, 2011).

Here, deletion analysis of *P. aeruginosa parA* using the YTH system roughly assigned the homo- and hetero-oligomerization to the N-terminal part of the protein. Alanine scanning of charged and hydrophobic residues in the ParA region E67–L85 and analysis of ParA variants in the BACTH system clearly demonstrated the essentiality of this region for both ParA self-interactions and interactions with ParB. Residues M74, H79, Y82 and L84 were shown to mediate ParA dimerization, and mutations at these positions were defective in interactions with ParB, indicating that only dimeric forms of ParA may associate with ParB. ParA variants with alanine substitutions at positions E67, C68, L70, E72, F76, Q83 and L85 were still capable of dimerization in the BACTH system, but impaired in interactions with ParB, thus defining a patch likely specific for hetero-oligomerization.

**Fig. 5.**
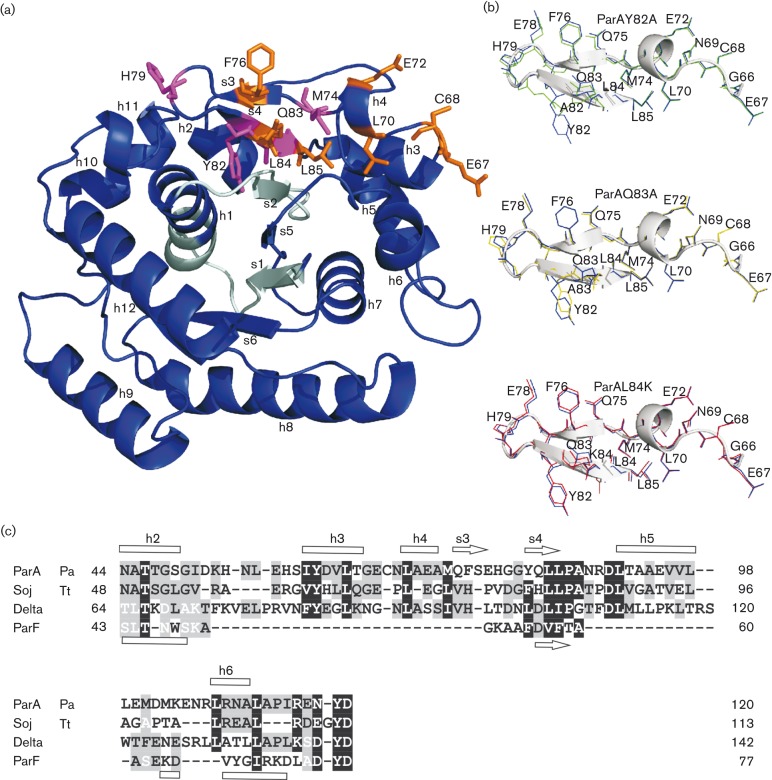
Model of the *P. aeruginosa* ParA monomer. (a) Model structure of ParA shown as a ribbon representation. The region of Walker motifs is highlighted in light blue. Amino acid residues important for interactions with ParB are shown as orange sticks; those playing roles in dimerization and interactions with ParB are shown as magenta sticks. Secondary structure elements marked according to the structure of Soj from *T. thermophilus* (Leonard *et al.*, 2005). (b) Overlaid structures of pseudo-hairpins in the WT and the most ‘distorted’ mutant derivatives of ParA. Structures forced by Y82A, Q83A and L84K substitutions are shown in green, yellow and red, respectively, whereas WT residues are shown in blue. (c) Alignment of *P. aeruginosa* (Pa) ParA fragment (44–120 aa) with Soj [*T. thermophilus* (Tt)], Delta (pSM19035) and ParF (TP228). The alignment was prepared using cobalt (Papadopoulos & Agarwala, 2007), HHpred (Söding *et al.*, 2005) and fatcat (Ye & Godzik, 2003) servers, and then corrected manually. Secondary structures are indicated according to Protein Data Bank entries 1WCV and 4DZZ for Soj and ParF, respectively. The *P. aeruginosa* ParA interactive pseudo-hairpin loop encompassed amino acid residues between G66 and L85. h, Helix; s, sheet.

The 3D model of *P. aeruginosa* ParA (Fig. 5a) built on the basis of crystallographic data for the representative of the chromosomal ParA subfamily, Soj of *T. thermophilus* (Leonard *et al.*, 2005), demonstrated that the region defined experimentally as important for dimerization and interactions with ParB encompasses two β-sheets folded into a pseudo-hairpin between helix 3 and 5 (Fig. 5c). The residues important for interactions with ParB are located at the external part of the loop, consistent with the idea that this part of the protein may be involved in partner binding. Comparison of predicted 3D structures of WT and mutant ParAs did not indicate any important conformational changes, with root-mean-square deviations for the backbone atoms of regions 1–65 and 86–254 being ≤0.1 Å. Only three mutant proteins, ParAY82A, ParAQ83A and ParAL84K, showed slight distortions in the region G66–L85 of the pseudo-hairpin structure as presented in Fig. 5(b). However, the introduced modifications did not affect loop folding, and were strictly limited to the substituted and adjacent residues. Two proteins, ParAQ83A and ParAL84A, were tested for ATPase activity, and proved capable of ATP hydrolysis at a comparable rate to the WT ParA (Fig. 3). This strongly suggested no major changes in the structures of mutant proteins.

The ATPase activity of *P. aeruginosa* ParA is in the range of 60–70 mol Pi released h^−1^ (mol protein)^−1^, much higher than observed for other proteins of this subfamily, e.g. 0.3−1.8 mol Pi released h^−1^ (mol protein)^−1^ (Ah-Seng *et al.*, 2009; Batt *et al.*, 2009; Leonard *et al.*, 2005; Schofield *et al.*, 2010). The ATPase activity of the dimerization-deficient ParAL84K suggests that impairment of self-interactions does not affect its enzymic function. Further studies are required to define the role of ATP binding in the ParA dimerization process.

Apart from the 3D structure of Soj from *T. thermophilus*, crystallographic data for three plasmid representatives of partitioning Walker-type ATPases are available: ParA of P1 plasmid prophage, Delta of pSM19035 and ParF of pTP228 (Dunham *et al.*, 2009; Pratto *et al.*, 2008; Schumacher *et al.*, 2012). Only Delta protein of pSM19035 (Pratto *et al.*, 2008) contains a pseudo-hairpin structure similar to the two chromosomal homologues (Fig. 5c). We reported previously that a Delta variant with alanine substitutions of two hydrophobic residues corresponding to L84 and L85 in ParA was impaired in dimerization and interactions with the Omega partner (Dmowski & Jagura-Burdzy, 2011). This suggests that some members of the ParA family of deviant Walker-type ATPases may rely on the same interface of interactions with their partners.

Screening of the database with the amino acid sequence from I60 to N110 of *P. aeruginosa* ParA encompassing the region we have identified as important for ParA–ParB interactions demonstrated that the *Pseudomonas* clade is separated from other species (the closest homologues are encoded by betaproteobacteria). The alignment of these regions originated from predicted ParAs encoded by the 35 *Pseudomonas* genomes clearly discriminated between different *Pseudomonas* species (Fig. S3), suggesting that observed subtle changes may determine the extent/lack of cross-reactivity between Par systems.

The PAO1161 *parA84* mutant (encoding a ParA impaired in dimerization and interactions with ParB) showed defects in growth rate, nucleoid segregation, ParB localization, and swarming and swimming motility comparable to the *parA*-null mutant (Fig. 4). Immunofluorescence microscopy revealed that ParB can form one to four foci in cells of PAO1161 *parA84*; however, these are mislocalized in the majority of cells, positioned asymmetrically, close to the cell poles and paired (Fig. 4d). The PAO1161 *parA84* mutant produces anucleate cells 300-fold more frequently than the WT strain. Despite such an increase in production of anucleate cells, the vast majority of the cells can survive and segregate their chromosomes, although with elongated division times. To distinguish between the role of ParA alone and in complex with ParB, we also constructed the PAO1161 *parA67* mutant encoding a ParA that can dimerize, but is impaired in interactions with ParB in the tests applied. Phenotypic characterization of the mutant demonstrated only mild changes when compared with the WT strain. No defects in the growth rate and no increase in the production of anucleate cells were observed for this mutant, but the noticeable impairment of swarming ability as well as evident coupling of ParB foci in the polar regions of the cells clearly indicated the role of ParA–ParB interactions in these processes. Whereas it is well established that ParA–ParB interactions are crucial for their function in the separation of *ori* domains, the unique role of both Par proteins in swimming and swarming of *P. aeruginosa* cells is not fully understood (Bartosik *et al.*, 2009; Lasocki *et al.*, 2007). In our search for ParB partners we have identified a protein affecting expression of ‘motility’ operons (manuscript in preparation). It is feasible that ParB interactions with this partner are influenced by association with ParA. The stronger mutant phenotype of PAO1161 *parAL84* than PAO1161 *parAE67* suggests that the dimerization/polymerization of ParA may be more important than ParA–ParB interactions in the biology of *P. aeruginosa*. However, we cannot exclude the possibility that the weak phenotype of the ParAE67A mutant protein is because in live cells it is still capable of limited interactions with ParB.

Our transcriptomic studies (Bartosik *et al.*, 2014) on *parA* and *parB* mutants of *P. aeruginosa* indicated major changes in gene expression patterns in both mutant strains. Despite the large overlap between the ParA and ParB regulon in *P. aeruginosa*, there are also clusters of genes regulated by ParA and ParB separately, which fits with the data presented here. Further studies are therefore required to explain the mechanisms that Par proteins use to regulate specific cell functions.
